# A miRNA signature in endothelial cell-derived extracellular vesicles in tumor-bearing mice

**DOI:** 10.1038/s41598-019-52466-1

**Published:** 2019-11-14

**Authors:** James V. McCann, Amber Liu, Luca Musante, Uta Erdbrügger, Joanne Lannigan, Andrew C. Dudley

**Affiliations:** 1 Department of Cell Biology & Physiology, University of North Carolina at Chapel Hill, Chapel Hill, NC 27599 USA; 2 Department of Microbiology, Immunology, and Cancer Biology, The University of Virginia, Charlottesville, VA 22908 USA; 3 Department of Medicine, Division of Nephrology, The University of Virginia, Charlottesville, VA 22908 USA; 4 Flow Cytometry Core, The University of Virginia, Charlottesville, VA 22908 USA; 5 Emily Couric Cancer Center, The University of Virginia, Charlottesville, USA

**Keywords:** Tumour angiogenesis, Breast cancer

## Abstract

Extracellular vesicles (EVs) play important roles in tumor progression by altering immune surveillance, promoting vascular dysfunction, and priming distant sites for organotropic metastases. The miRNA expression patterns in circulating EVs are important diagnostic tools in cancer. However, multiple cell types within the tumor microenvironment (TME) including cancer cells and stromal cells (e.g. immune cells, fibroblasts, and endothelial cells, ECs) contribute to the pool of circulating EVs. Because EVs of different cellular origins have different functional properties, auditing the cargo derived from cell type-specific EVs in the TME is essential. Here, we demonstrate that a murine EC lineage-tracing model (Cdh5-Cre^ERT2^:ZSGreen^l/s/l^ mice) can be used to isolate EC-derived extracellular vesicles (EC-EVs). We further show that purified ZSGreen^+^ EVs express expected EV markers, they are transferable to multiple recipient cells, and circulating EC-EVs from tumor-bearing mice harbor elevated levels of specific miRNAs (e.g. miR-30c, miR-126, miR-146a, and miR-125b) compared to non tumor-bearing counterparts. These results suggest that, in the tumor setting, ECs may systemically direct the function of heterotypic cell types either in the circulation or in different organ micro-environments via the cargo contained within their EVs.

## Introduction

Extracellular vesicles (EVs) are abundant 30–1000 nm membrane-bound particles found in virtually all biological fluids. They are further classified as exosomes (derived from multi-vesicular bodies, MVBs) or microvesicles (MVs, shed or budded from plasma membranes)^1^. EVs have emerged as important vectors for transferring proteins and nucleic acids between heterotypic cell types; particularly in cancer^2^. Although the abundance and stoichiometry of RNAs contained within EVs has been questioned, circulating EVs probed for their RNA cargo have provided diagnostic information to predict, for example, tumor recurrence, metastasis, and progression-free survival^3^. Cancer cell-derived EVs, enriched in proteins, lipids, and nucleic acids “re-educate” different cellular recipients to promote tumor growth, metastasis, and drug resistance through diverse mechanisms^4–9^. While cancer cell-derived EVs have been well-characterized, few studies have explored the content and function of EVs secreted from auxiliary cell-types found within the tumor microenvironment (TME). To do so, new genetic tools are needed to label, isolate, and audit EVs derived from cancer cells and other supportive cell types found in solid tumors; especially *in vivo*. It was recently shown that the ZSGreen fluorophore (excitation max, 493 nm; emission max, 505 nm), derived from Zoanthus reef coral and modified for high solubility, bright emission, and rapid chromophore maturation is exported via EVs. In a melanoma model, ZSGreen^+^ vesicles from melanoma cells were observed in the draining lymph nodes and were engulfed by dendritic cells^10^. However, no studies have taken advantage of ZSGreen’s tropism for EVs to study the miRNA cargo in circulating EVs in the cancer setting *in vivo*. Endothelial cells (ECs) are a potentially enriched source for circulating EVs because they are in direct contact with the circulation. This places ECs in a unique position to transfer information via EVs systemically (i.e. luminal secretion) or to nearby cell types within the complex TME. Our lab routinely uses Cdh5-Cre^ERT2^:ZSGreen^l/s/l^ mice (herein referred to as EC^iZSGreen^ mice) as a strategy to study the differentiation and functions of ECs in tumors^11,12^; we therefore hypothesized that these mice could be used as a genetic tool to isolate and characterize EC-EVs in the cancer setting.

## Methods

### Laboratory mice and ***in vivo*** studies

Cdh5-Cre^ERT2^ mice were crossed with Ai6 ZSGreen reporter mice to generate EC^iZSGreen^ mice. Cdh5-Cre^ERT2^ mice were generated by Ralf Adams (Max Plank Institute for Molecular Biomedicine, Munster, Germany. Ai6 ZSGreen mice were purchased from The Jackson Laboratory at 7 weeks of age. LSL-KRAS^G12D/+^ p53^fl/fl^ Lkb1^fl/fl^ mice were provided by Dr. Chad Pecot (UNC Chapel Hill). Cdh5-Cre^ERT2^ and Ai6 ZSGreen mice were all on a C57BL/6 background. LSL-KRAS^G12D/+^ p53^fl/fl^ Lkb1^fl/fl^ mice were on a 129/s4 background. Tumor studies and EV or EC isolations were performed on 8- to 10-week-old mice with a mean weight of 18–22 g. Age-matched mice, generated from breeding pairs, were randomly allocated to each experimental or control group. Tamoxifen induction was carried out as previously described^12^.

### Cell lines

All primary ECs were isolated from normal or tumor tissues and grown in a defined media as previously described^13^.

### Tumor studies in mice

EC^iZSGreen^ mice were used in mammary tumor studies. E0771 mammary tumor cells were orthotopically injected into the mammary fat pad as previously described by us^12^. Lung tumors were induced and ECs were isolated as described previously^12^. For total EV and EC-EV quantification, mammary tumors were allowed to grow to 0.8–1.0 cm^3^ before plasma was harvested for EV isolation.

### Western blot analysis

Proteins on Western blots were detected using the following antibodies raised against: CD9 (sc-13118), CD81 (sc-166029), CD63 (sc-15363), GAPDH (Cell Signaling, 5174), TSG101 (Sigma, T5701), and ZSGreen (Clonetech, 632598).

### Immunofluorescence

ZSGreen^+^ ECs were grown on glass chamber slides, fixed with methanol, permeabilized with 0.1% Saponin in blocking buffer (1% BSA + 5% Goat Serum), blocked 1 hr at room temperature, incubated overnight in anti-Lamp1 antibody (ab25245), and then incubated 1:200 with goat anti-rat 594 antibody for 1 hr at room temperature (Invitrogen, A-11007).

### EV isolation (***in vitro***)

ECs were grown in 1% exosome-depleted FBS (System Biosciences LLC) in LG-DMEM for 48 hrs and then subjected to differential centrifugation. Conditioned media (CM) was spun at 250 g for 5 minutes, 2,000 g for 10 minutes, and then 100,000 g for 1.5 hrs. All centrifugation steps were performed at 4 C on a Beckman Coulter Optima LE-80K Ultracentrifuge with SW-41TI rotor in Beckman Coulter thin wall, polypropylene centrifuge tubes. The EV pellet was resuspended in Dulbecco’s phosphate buffered saline (DPBS) and stored at −80 C until characterization or immediately lysed with RIPA buffer or RNA lysis buffer. Cells from culture dishes were counted to normalize the number of EV-secreting cells to the number of EVs collected and to ensure that cells were similarly viable. Media only was also centrifuged and run as a control.

### EV isolation (***in vivo***)

Blood was collected from mice via cardiac puncture and spun at 4,000 g for 10 mins at room temperature to isolate platelet-free plasma. Plasma was removed and spun again at 4,000 g to ensure no cell or platelet contamination. The remaining platelet-free plasma was diluted in 10 mL of DPBS and spun at 100,000 g for 1.5 hrs at 4 C.

### EV quantification

Size and concentration of EVs isolated from CM or plasma was determined using ZetaView NTA. All samples were diluted in water (1:250 to 1:1000 for CM and 1:250 to 1:5 for plasma). EVs were measured both in bright field (total) and using a 488 nm filter. Samples were read alongside buffer only controls to ensure no EV contamination from the buffer.

### RNA isolation and qPCR

RNA was isolated from cells and EVs using the Zymo Research Quick-RNA Microprep kit. miRNA Taqman primers were ordered from Life Technologies. cDNA was generated as previously described^12^. mRNA qPCR was run with Maxima Sybr Green and miRNA qPCR was run with TaqMan Universal Master Mix II. All qPCR samples were run in triplicate on an Applied Biosystems Quant Studio 6 instrument. mRNA was normalized to GAPDH, cell lysate miRNA was normalized to snoRNA234, and EV miRNA was normalized to miR-16.

### Nanostring array

RNA was purified as described above from control or tumor-bearing mice using EVs isolated from plasma. RNA samples were submitted to Nanostring (Seattle, WA) for the RNA array. Analysis of the Nanostring array was done using the nCounter software package. Background subtraction was performed to account for false positives. Positive controls and code-set content (house keeping gene) normalization was performed using the nCounter on all samples.

### Cryo-EM

EVs were isolated from CM as described above and resuspended in 20 uL of DPBS. EVs were then imaged using a Tecnai F20 Twin emission electron microscope at the Molecular Electron Microscopy Core at UVA.

### Flow cytometry

FACS analysis of isolated EVs from cell culture or plasma was performed on a BD Influx Cell Sorter. Buffer only was run prior to each sample to measure background and to ensure there was no contamination between samples. Samples were run with 70 uM nozzle at 30 psi with a drop frequency of 64. ZSGreen was detected with a band-path filter of 540/40 with a threshold of 0.65.

### ImageStream X analysis of EVs and cells

ImageStream X flow cytometry of isolated EVs was performed on an Amnis ImageStream X Mark II according to methods previously described^14–16^. ZsGreen fluorescence was excited with a 488 nm laser at 200 mW and emission collected using a 480–560 nm bandpass filter (Ch02). Buffer only was run prior to each sample (for the same acquisition time) to measure background and to ensure there was no contamination between samples. Control and EV-treated cells were analyzed after 48 hrs of incubation as described for EV transfer experiments.

### EV transfer (***in vitro***)

EVs harvested as described above were resuspended in exosome-depleted media described above and allowed to incubate for 48 hrs. Cells were then harvested for FACS or ImageStreamX.

### Statistical analysis

All data were analyzed using Student’s t-test and statistical significance is indicated with an asterisk (p < 0.05).

### Study approval

All animal experiments were carried out in accordance with and under approval of the Institutional Animal Care and Use Committees (IACUC) of the University of Virginia and the University of North Carolina at Chapel Hill. Both universities are accredited by the Association for the Assessment and Accreditation of Laboratory Care (AAALAC) International and follow the Public Health Service Policy for the Care and Use of Laboratory Animals. Animal care was provided in accordance with the procedures outlined in the Guide for the Care and Use of Laboratory Animals and protocols were approved by UNC Chapel Hill or the University of Virginia Animal Care and Use Committees.

## Results

### ECs isolated from EC^iZSGreen^ mice secrete ZSGreen^+^ EVs that co-localize with endosomal Lamp1, they express EV markers, and they are transferable to multiple cellular recipients

**Figure 1:**
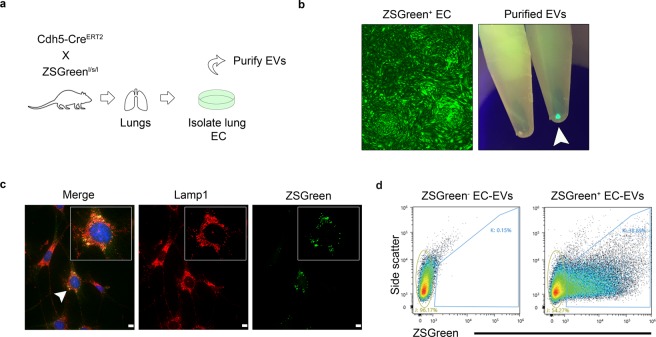
ECs isolated from EC^iZSGreen^ mice secrete ZSGreen^+^ EVs that are derived from endosomes. (**a**) Schematic of the experimental approach. (**b**) Isolated ZSGreen^+^ lung ECs maintain brilliant ZSGreen fluorescence *in vitro* (left panel). At right, purified EVs from ZSGreen^−^ ECs and ZSGreen^+^ ECs under a fluorescent lamp (488 nm) are shown. ZSGreen^−^ EC-EVs are shown at left, and at right are ZSGreen^+^ EC-EVs (white arrowhead). (**c**) Immunohistochemistry of the endosomal marker Lamp1 (red fluorescence) and ZSGreen demonstrating their co-localization. (**d**) FACS plots and gating of ZSGreen^+^ EVs from the culture medium of ZSGreen^+^ ECs.

**Figure 2:**
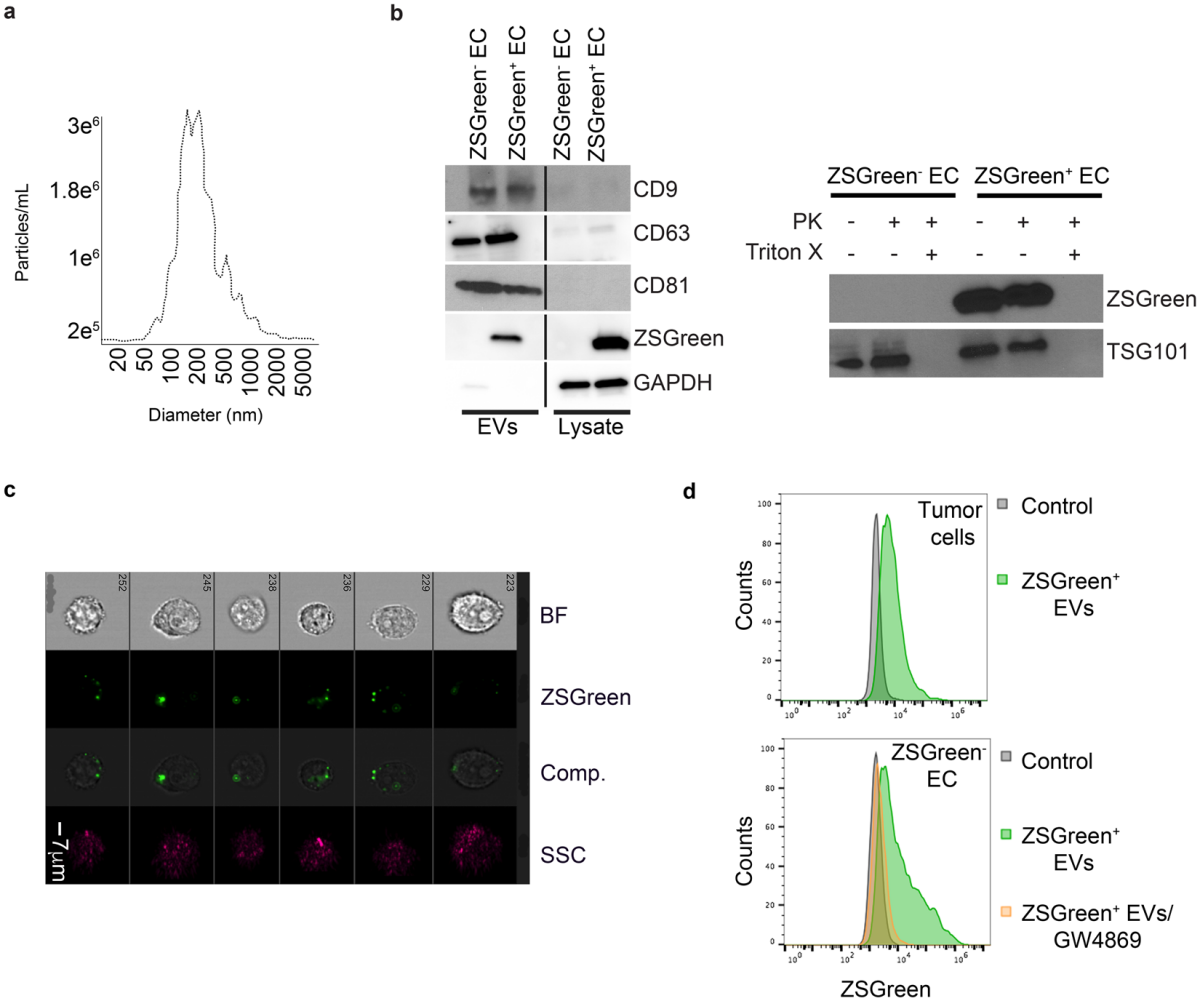
EVs from EC^iZSGreen^ mice express EV markers and are transferable to multiple cellular recipients. (**a**) ZetaView NTA plot of ZSGreen^+^ EVs detected using a 488 nm filter. (**b**) Western blotting for CD9, CD63, CD81, ZSGreen, and GAPDH in the indicated samples and ZSGreen or TSG101 western blot using the indicated fractions from ZSGreen^−^ ECs or ZSGreen^+^ ECs treated +/− proteinase K (PK) or +/− Triton X. (**c**) Bright field (BF) and fluorescent channel images from ImageStream X flow cytometry using murine E0771 mammary tumor cells incubated with ZSGreen^+^ EC-EVs. SSC indicates auto fluorescence which shows no overlap with ZSGreen signals, comp. = composite. (**d**) Representative FACS plots of E0771 mammary tumor cells spiked with ZSGreen^−^ EC-EVs (control) or ZSGreen^+^ EC-EVs (top). At bottom, FACS plot of ZSGreen^−^ ECs spiked with ZSGreen^+^ EC-EVs from EC cultures that were treated 24 hours prior with GW4869.

We crossed Cdh5-Cre^ERT2^ mice with Ai6 ZSGreen reporter mouse to generate EC^iZSGreen^ mice (Fig. 1a). We then isolated primary lung ECs following our previously published protocols^12^. EC purity was confirmed by expression of ZSGreen and expression of bona fide EC markers (Fig. 1b and data not shown). To determine if ZSGreen^+^ EVs were secreted from ECs, we subjected the conditioned media to high speed (x100,000 g) differential ultracentrifugation following established protocols for purifying EVs according to MISEV (minimal information for studies of EVs) guidelines^17^. Strikingly, the purified EVs brightly fluoresced when exposed to a 488 nm wavelength lamp, suggesting ZSGreen protein is exported into EVs (Fig. 1b). Since EVs can be derived from endosomes, we sought to determine whether ZSGreen might localize with the endosomal marker Lamp1. Using immunofluorescence microscopy of methanol-fixed ECs, we found that ZSGreen was distributed throughout the EC cytoplasm but was also present within puncta that co-localized with Lamp1 suggesting that ZSGreen is indeed directed into endosomes that could precede its export via EVs (Fig. 1c)^18^. To test this possibility, we used fluorescence activated cell sorting (FACS) to isolate ZSGreen^+^ EC-EVs from conditioned media and then used the Particle Metrix Zetaview Nanoparticle Tracking Analysis (NTA) system equipped with 488 nm laser to determine particle size. Using this approach, we found that ZSGreen^+^ EVs had a median size of 111.6 ± 86.6 nm which is in good accord with their designation as EVs/exosomes or small EVs (Fig. 1d and Fig. 2a)^19^. Additionally, FACS-isolated ZSGreen^+^ particles were found to have a median diameter of ~75–100 nm using qNano (tunable resistive pule sensing, data not shown). To visualize and confirm the presence of ZSGreen in ZSGreen^+^ EC-EVs, we used ImageStream X flow cytometry and found that fluorescent EVs were visible under the green fluorescent channel but not by bright field luminescence indicating fidelity of the detected signal as previously demonstrated by us (Fig. S1a–c)^15^. We then characterized the protein cargo of ZSGreen^+^ EVs and found they express multiple EV markers (e.g. CD9, CD63, and CD81) and that ZSGreen is housed within EVs indicated by its protection from proteinase K (PK) treatment similar to the Endosomal Sorting Complex Required for Transport (ESCRT) protein TSG101 (Fig. 2b)^20^. No ZSGreen or TSG101 were detected when EVs were solubilized with Triton X, as expected. Finally, we tested whether ZSGreen^+^ vesicles could be transferred to unlabeled recipient cells. We isolated ZSGreen^+^ EVs from conditioned media and fed them to unlabeled ZSGreen^−^ EO771 mammary tumor cells. Using ImageStream X flow cytometry, we found that cancer cells readily uptake ZSGreen^+^ EC-EVs indicated by a punctate pattern of intracellular fluorescence (Fig. 2c). These results were confirmed using E0771 tumor cells that were pulsed with ZSGreen^+^ EC-EVs, stringently washed, and then analyzed using FACS (Fig. 2d). Consistent with ZSGreen packaging within EVs/exosomes, the uptake of labeled EVs was greatly reduced when donor ZSGreen^+^ ECs were treated with the EV biogenesis inhibitor GW4869 - although we cannot rule out the possibility that inhibition of ceramide by GW4869 does not impact additional cellular properties important for sub cellular particle trafficking/secretion (Fig. 2d)^2^. Taken together, these data support the concept that ZSGreen^+^ ECs secrete ZSGreen^+^ vesicles that fall within the size range of EVs, they express bona fide EV markers, and they are freely transferable to homotypic or heterotypic cell types.

### Purified ZSGreen^+^ EVs from tumor-bearing EC^iZSGreen^ mice secrete ZSGreen^+^ EVs into plasma that are enriched in specific miRNAs

**Figure 3:**
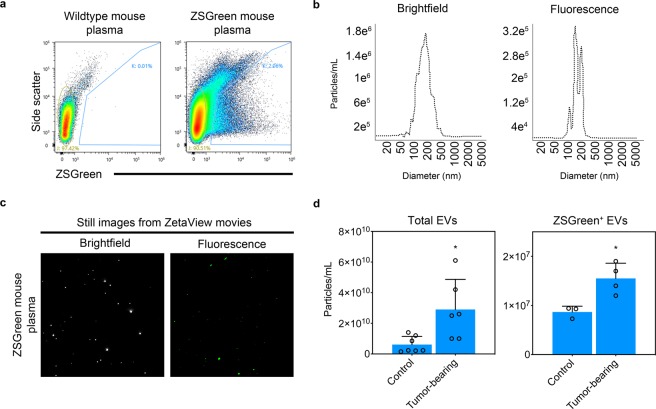
EC^iZSGreen^ mice secrete ZSGreen^+^ EC-EVs into plasma. (**a**) FACS plots using EVs isolated from wildtype (ZSGreen^−^) or EC^iZSGreen^ mice. (**b**) ZetaView NTA plots of bright field and fluorescence using EVs isolated from the plasma of EC^iZSGreen^ mice. (**c**) Still images from ZetaView NTA movies using EVs isolated from EC^iZSGreen^ mice. (**d**) Quantification of total and fluorescent ZSGreen^+^ EVs from control versus mammary tumor bearing mice using ZetaView NTA (EVs were pooled from n = 3 mice; 3–7 biological replicates). Results were analyzed using Student’s t-test, *p < 0.05.

To test whether ZSGreen^+^ EVs were detectable in mouse plasma *in vivo*, we isolated EVs from platelet-free plasma from EC^iZSGreen^ mice. Using FACS, we found that ZSGreen^+^ EVs were detectable using a 488 nm laser and were the expected EV size as determined by NTA (~187 +/− 129.2 nm for bright field [total] and 177 +/− 110.6 nm for fluorescence [EC-derived]) (Fig. 3a,b). We took advantage of the NTA 488 nm fluorescent filter to measure total circulating ZSGreen^−^ EVs versus EC-derived (ZSGreen^+^) EVs in control mice or in mice bearing orthotopic mammary tumors. The results showed that mice bearing mammary tumors had a 4.8-fold increase in the total numbers of circulating vesicles (2.9e^10^ ± 1.9e^10^ particles/ml versus 6.1e^9^ ± 5.3e^9^ particles/ml) and a 1.8-fold increase in ZSGreen^+^ EC-EVs (1.6e^7^ ± 3.1e^6^ particles/ml versus 8.7e^6^ ± 1.2e^6^ particles/ml) compared to their non tumor-bearing counterparts (Fig. 3c,d and Fig. S2a,b) - also see accompanying supplemental movies. These results suggest that solid tumors invoke an increase in total circulating EVs and also an increase in circulating EVs that are specifically derived from the vasculature.

**Figure 4:**
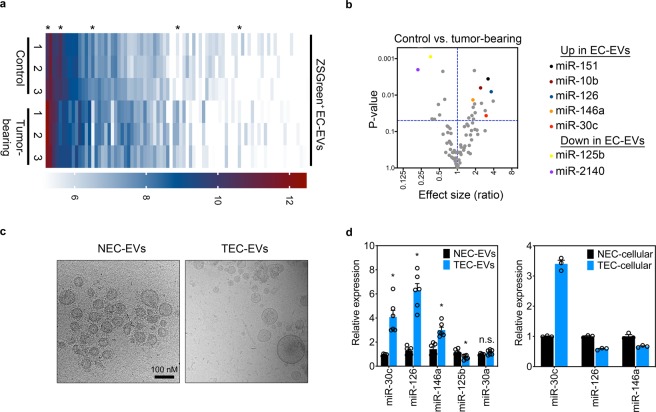
EC-EVs from tumor-bearing mice are enriched in specific miRNAs. (**a**) Nanostring miRNA array heat map using FACS-isolated ZSGreen^+^ EVs from control versus mammary tumor-bearing mice (n = 3 mice/group). Asterisks indicate a few differentially-expressed miRNAs of interest. (**b**) Volcano plot of miRNAs detected in ZSGreen^+^ EC-EVs from control versus mammary tumor-bearing mice. Selected miRNAs of interest that were determined to be statistically significant are indicated at far right. (**c**) Cryo-EM images of EVs isolated from conditioned media of normal mammary gland (NECs) or C3-TAg mammary tumors (TECs). The scale bar applies to both panels. (**d**) qPCR analysis of selected miRNAs in the indicated EVs (n = 2 individual samples tested in triplicate) or in cellular fractions (n = 3). Results were analyzed using Student’s t-test, *p < 0.05.

Compared to normal cell types, cancer cells are known to aberrantly express several miRNAs (and proteins); therefore, we asked whether miRNA cargoes within circulating ZSGreen^+^ EC-EVs showed differential expression patterns using control versus mammary tumor-bearing mice. Using a Nanostring miRNA array, we screened ~600 miRNAs in ZSGreen^+^ EC-EVs with ~80 landing above the detection limits of the assay; moreover, several of these miRNAs were either up- or down-regulated in tumor-bearing mice compared to their control, age- and sex-matched counterparts (Fig. 4a,b and Fig. S2c). A complete list of the differentially-expressed miRNAs uncovered in EC-EVs using control versus tumor-bearing mice is shown in the online data supplement. Interestingly, several miRNAs that were significantly elevated in EC-EVs from tumor-bearing mice have recently been shown to be important during tumor progression. For example, we recently showed tumor-suppressive roles for miR-30c in the endothelium^12^. Similarly, miR-146a, that we found to be elevated in ZSGreen^+^ EC-EVs, was recently shown to be immune suppressive in the melanoma tumor microenvironment^21^. miR-126, miR-10b, and miR-151 were all shown to be elevated or have diagnostic utility in the serum of patients with a variety of cancer types and subtypes; but none of these studies have demonstrated a potential EC origin for these miRNAs in these patients^22–24^.

We next used primary ECs isolated from C3-TAg mammary tumors (tumor endothelial cells, TECs) to assess their EC-EV miRNA signatures *in vitro*^12^. The EVs isolated from conditioned media showed a characteristic lipid bilayer and were of an expected EV size as measured by cryo-electron microscopy (Fig. 4c). Using qPCR, we could confirm selective increases in miR-30c, miR-126, and miR-146a in EVs derived from TECs versus normal ECs (NECs) whereas miR-125b was reduced; consistent with the Nanostring array (Fig. 4d). Notably, only miR-30c was elevated in the cellular fraction suggesting there may be selective export/enrichment of specific miRNAs into EVs. Using NTA with C3-TAg TECs and KRAS^G12D^ lung TECs^12^, we found that EVs isolated from NECs and TECs were of similar size; however, mammary TECs secreted 7.4 times as many EVs per cell (2.8e^4^ ± 1.28e^4^ versus 3.3e^3^ ± 3.3e^2^) and lung TECs secreted 2.4 times as many EVs per cell (3.3e^4^ ± 2.9e^3^ versus 1.4e^4^ ± 3.6e^3^) compared to their normal counterparts (Fig. S2d–g). These data support the possibility that EV production is increased in the vasculature in the cancer setting leading to an enrichment of total EC-EV miRNA (and protein) cargo in the systemic circulation.

## Discussion

EVs have emerged as important vectors for exchanging information between cancer cells and cancer-associated stromal cells. The cargo contained within EVs mirrors the cell of origin; therefore, it is anticipated that EVs derived from different cell types can differentially impact their cellular recipients. However, genetic tools to isolate and characterize EVs derived from different cell types in the cancer setting are lacking. Typically, purified EVs are labeled with a lipophilic dye and then re-injected into laboratory mice. Genetic approaches to endogenously label EVs, without *in vitro* manipulation, will advance our understanding of EV biology in general and help to clarify the cell type-specific roles of EVs in the complex TME. The model we present herein has taken advantage of the selective export of ZSGreen into EC-EVs using EC-specific Cre-driver mice. This approach is easily adaptable to study EVs of any cell-of-origin by substitution with different lineage-specific Cre-drivers; thus, our model is versatile and could be used to examine the EV content of multiple cell types (e.g. immune cells or fibroblasts) found within solid tumors.

There are a few limitations in this study that may impact our analysis and will require resolution. For example, while we have shown that ZSGreen protein is detected within EVs/exosomes, we also find membrane/cytoplasmic ZSGreen fluorescence in cultured ZSGreen^+^ ECs. It is presumed therefore that other sub cellular particles >150 nm will contain both ZSGreen and miRNAs but these particles would have to be purified using different approaches (e.g. differential centrifugation and sucrose gradients); similarly, the quantification of ZSGreen^+^ vesicles using NTA could include co-purified heterogeneous ZSGreen^+^ particles also found in mouse plasma, or particles derived from a small population of Cdh5^+^ hematopoietic cells, potentially skewing the ratio of total circulating EVs:EC-derived EVs^25^. In our *in vitro* assays, there also appear to be Lamp1^+^ endosomes that are ZSGreen^−^resulting in an underestimation of EC-EVs/exosomes in the circulation and identification of only a partial miRNA signature in EC-EVs. Each of these limitations could be addressed using more sophisticated mouse models where an EV marker (e.g. CD63 or CD9) is fused to a fluorescent reporter enabling the specific isolation of bona fide endosome-derived EVs. Finally, it is not clear why ZSGreen is deposited into sub cellular particles (i.e. endosomes) and then ultimately exported via EVs. Green fluorescent protein (GFP) is also known to be evicted from cells via a non-classical pathway following its over-expression, improper folding, and localization to aggresomes; however these mis-folded proteins typically no longer fluoresce limiting their utility as tracers to identify and isolate EVs from specific cell types *in vivo*^26^.

Despite these limitations, our results support the concept that the vasculature is an important source for circulating EVs and that specific miRNAs packaged within EC-EVs have the potential to systemically reprogram different cellular recipients. For example, although the uptake of cancer cell-derived EVs by the endothelium has been documented, cancer cells proximal to the vasculature are also free to engulf vascular-derived EVs leading to gene expression changes or alterations in their behavior/function^4,27^. Since ECs are uniquely positioned within solid tumors (in contact with the circulation at the luminal surface and other cell types including cancer cells at the abluminal surface) we view the tumor endothelium as a “sentinel” that could readily exchange information with other cell types via their EV payloads in both primary tumors and in sites of metastasis. A good example are circulating innate and adaptive immune cells, or those immune cells found within draining lymph nodes, that could be reprogrammed following the uptake of proteins or nucleic acids derived from dysfunctional ECs found in solid tumors.

## Supplementary information

Supplementary figure legends

Supplementary figures

Supplementary video 1- Total plasma EVs

Supplementary video 2-ZSGreen plasma EVs

Supplementary miRNA nanostring array
