# Supplementary material for: A miRNA signature in endothelial cell-derived extracellular vesicles in tumor-bearing mice
Source: Sci Rep. 2019 Nov 14;9:16743. doi: 10.1038/s41598-019-52466-1 (PMC6856062; doi:10.1038/s41598-019-52466-1)
Supplement: Supplementary file 1 — Supplementary figure legends [file 41598_2019_52466_MOESM1_ESM.docx]

**Supplementary Fig 1: Representative ImageStream X analysis of ZSGreen^+^ EC-EVs.** (a) Intensity and scatter analysis of ZSGreen^+^EVs. (b) Gating strategy of ZSGreen^+^ EC-EVs using ImageStream X. (c) Representative bright field (BF) and fluorescent images of singular ZSGreen^+^ EVs.

**Supplementary Fig 2: ZetaView NTA and qPCR analysis of ZSGreen^+^ EVs.** (a) Bright field ZetaView NTA plots of total circulating EVs from plasma using E0771 mammary tumor-bearing mice. (b) Fluorescence ZetaView NTA plot of ZSGreen^+^ EVs, same as in “a”. (c) qPCR analysis to validate selected miRNAs from the Nanostring array using the indicated samples (n=3, assayed in triplicate). (d and e) Representative ZetaView NTA plots of EVs isolated from the conditioned media using NECs or TECs (n=3 separate experiments). (f) Number of EC-EVs released per cell using mammary NECs versus C3-TAg TECs in vitro (n=3). (g) Number of EC-EVs released per cell using lung NECs versus KRAS^G12D^ TECs in vitro (n=3).

**Supplementary table legend:** A table of miRNAs from the Nanostring analysis that fell above threshold.

**Supplementary movies:** Representative NTA movies using plasma viewed under bright field or fluorescence channels. Total plasma EVs and ZSGreen^+^ EC-EVs are detectable and are shown.
