# Supplementary material for: A miRNA signature in endothelial cell-derived extracellular vesicles in tumor-bearing mice
Source: Sci Rep. 2019 Nov 14;9:16743. doi: 10.1038/s41598-019-52466-1 (PMC6856062; doi:10.1038/s41598-019-52466-1)
Supplement: Supplementary file 2 — Supplementary figures [file 41598_2019_52466_MOESM2_ESM.pdf]

Title: A miRNA signature in endothelial cell-derived extracellular vesicles in tumor-bearing mice

Authors: James V. McCann [1], Amber Liu [2], Luca Musante [3], Uta Erdbrugger [3], Joanne Lannigan [4], and Andrew C. Dudley [2,5]

Affiliations:

[1] Department of Cell Biology & Physiology, University of North Carolina at Chapel Hill, Chapel Hill, NC 27599, USA

[2] Department of Microbiology, Immunology, and Cancer Biology, The University of Virginia, Charlottesville, VA 22908, USA

[3] Department of Medicine, Division of Nephrology, The University of Virginia, Charlottesville, VA 22908, USA

[4] Flow Cytometry Core, The University of Virginia, Charlottesville, VA 22908, USA

[5] Emily Couric Cancer Center, The University of Virginia

Correspondence:

Dr. Andrew C. Dudley
Dept. of Microbiology, Immunology, and Cancer Cell Biology
The University of Virginia
Charlottesville, VA 22908
Tel: (434) 924-7766
Email: acd2g@virginia.edu

Key words: exosomes, extracellular vesicles, endothelial cells, tumor microenvironment, cancer, angiogenesis, miRNA

THERE ARE NO CONFLICTS OF INTEREST TO DISCLOSE

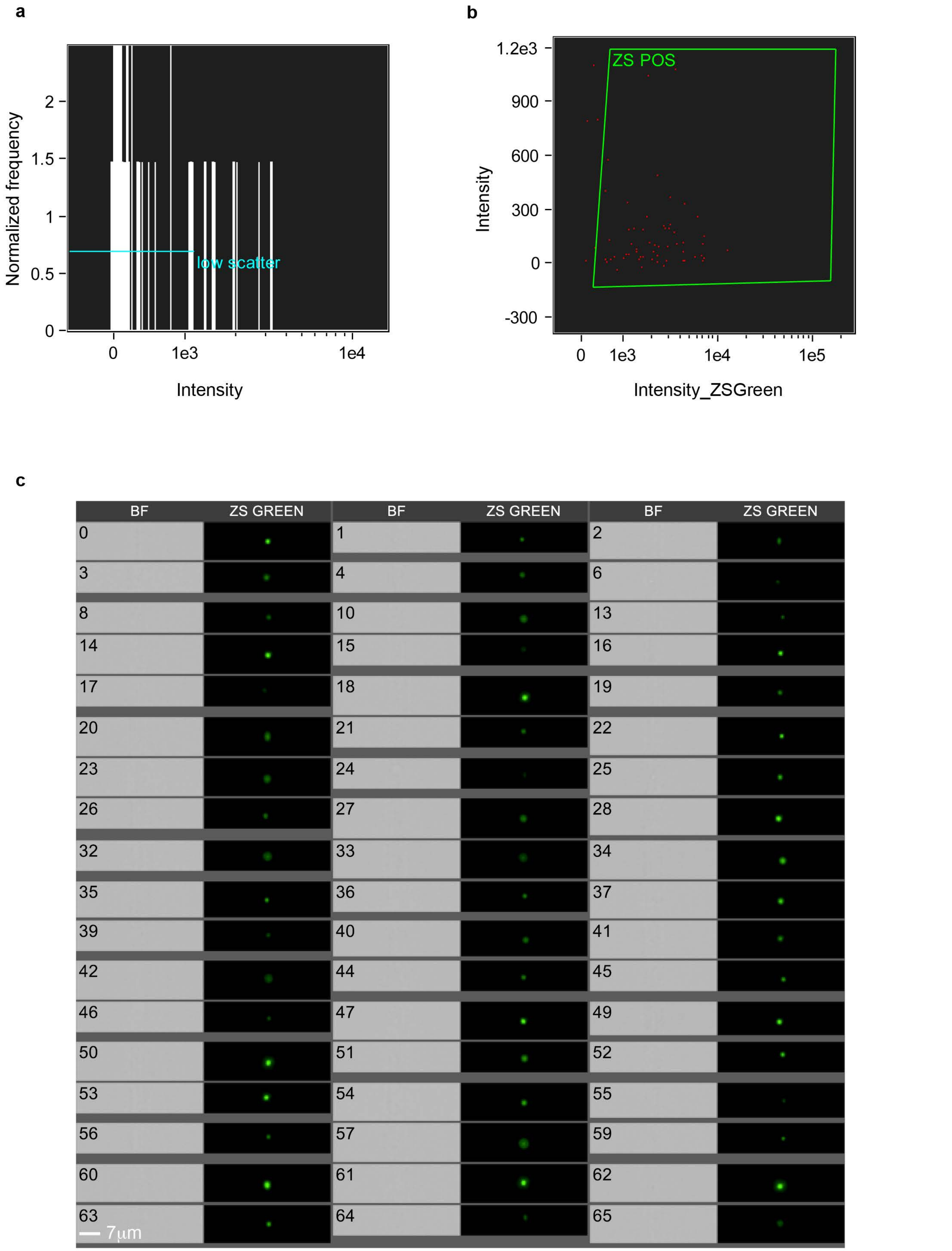
a
Normalized frequency
Intensity
0
1e3
1e4
0
0.5
1
1.5
2
low scatter
b
Intensity
Intensity_ZSGreen
1.2e3
900
600
300
0
-300
1e3
1e4
1e5
ZS POS
c
BF
ZS GREEN
0 1 2 3 4 6 8 10 13 14 15 16 17 18 19 20 21 22 23 24 25 26 27 28 32 33 34 35 36 37 39 40 41 42 44 45 46 47 49 50 51 52 53 54 55 56 57 59 60 61 62 63 64 65
7μm

Fig S2

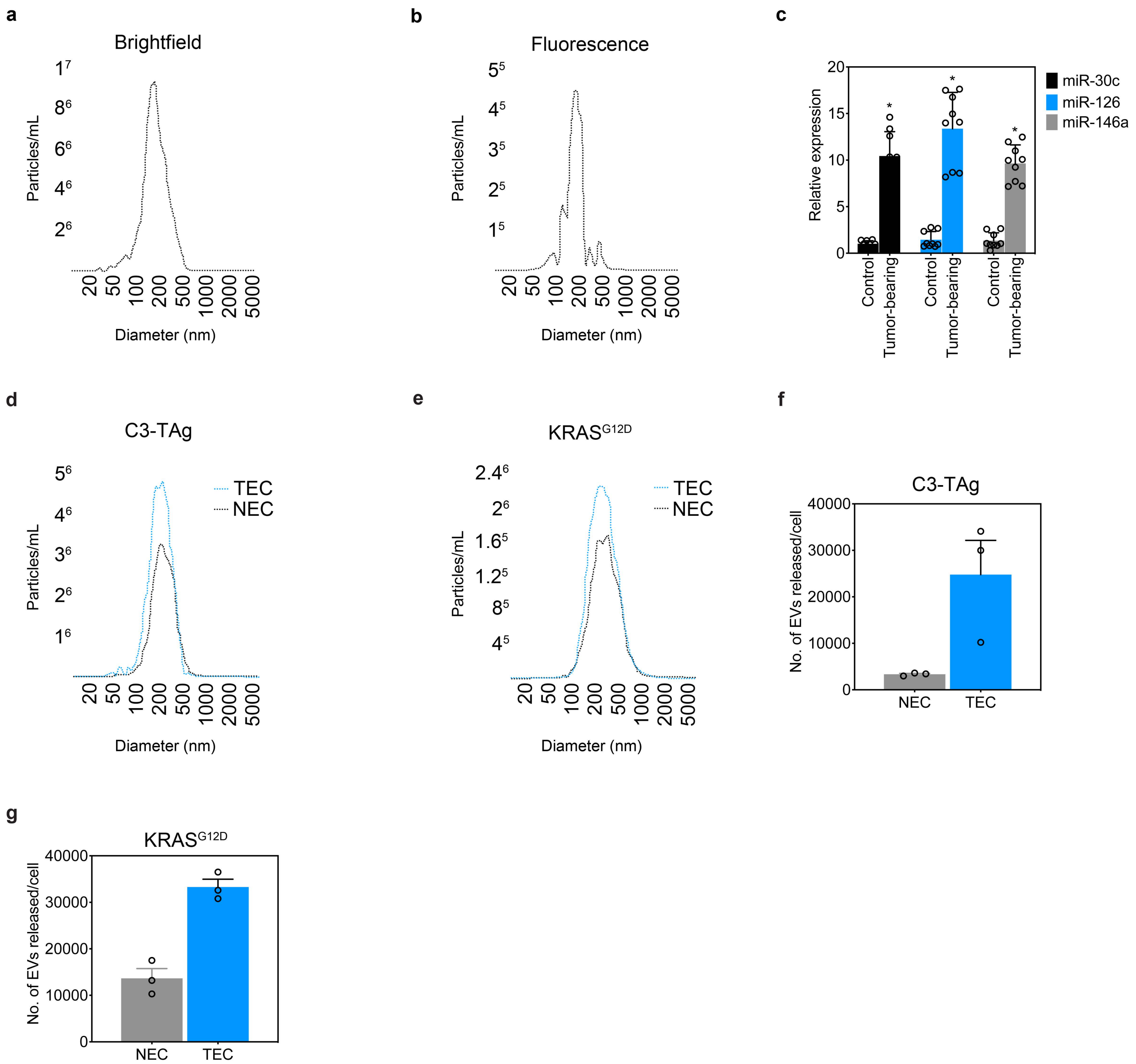

a
Brightfield
1^7
8^6
6^6
4^6
2^6
Particles/mL
20 50 100 200 500 1000 2000 5000
Diameter (nm)
b
Fluorescence
5^5
4^5
3^5
2^5
1^5
Particles/mL
20 50 100 200 500 1000 2000 5000
Diameter (nm)
c
Relative expression
20 15 10 5 0
miR-30c
miR-126
miR-146a
Control
Tumor-bearing
Control
Tumor-bearing
Control
Tumor-bearing
d
C3-TAg
5^6
4^6
3^6
2^6
1^6
Particles/mL
TEC
NEC
20 50 100 200 500 1000 2000 5000
Diameter (nm)
e
$KRAS^{G12D}$
2.4^6
2^6
1.6^5
1.2^5
8^5
4^5
Particles/mL
TEC
NEC
20 50 100 200 500 1000 2000 5000
Diameter (nm)
f
C3-TAg
No. of EVs released/cell
40000 30000 20000 10000 0
NEC
TEC
g
$KRAS^{G12D}$
No. of EVs released/cell
40000 30000 20000 10000 0
NEC
TEC
